# A phosphorous-based dendrimer targets mitochondria and normalizes the keratinocyte proliferation/differentiation balance to improve psoriasis

**DOI:** 10.1371/journal.pone.0343926

**Published:** 2026-03-31

**Authors:** Emily Clement, Ranime Jebbawi, Abdelouahd Oukhrib, Hélène Labie, Maëlys Le Moal, Séverine Fruchon, Stéphanie Cassel, Anne-Marie Caminade, Cédric-Olivier Turrin, Muriel Blanzat, Michel Simon, Rémy Poupot

**Affiliations:** 1 Univ Toulouse, CNRS, Inserm, Infinity, Toulouse, France; 2 CNRS, UMR 5623, Université de Toulouse, Toulouse, France; 3 Laboratoire de Chimie de Coordination (LCC), CNRS - Université de Toulouse, UPR 8241, Toulouse, France; UMass Chan Medical School Department of Medicine: University of Massachusetts Chan Medical School Department of Medicine, UNITED STATES OF AMERICA

## Abstract

Psoriasis vulgaris is a common chronic inflammatory skin disease associated with hyperproliferation and defective differentiation of keratinocytes. Despite new effective treatments using biologicals targeting key cytokines or their receptors, innovative therapeutic approaches remain to be discovered. Here, psoriasis-like imiquimod-induced skin lesions in mice were improved when topically treated with a nano-sized, phosphorus-based dendrimer capped with azabisphosphonate groups, so-called IMD-006, and previously known as ABP dendrimer. This effect was confirmed using *ex vivo* and *in vitro* human psoriasis models generated by adding T helper (Th)1-type and Th17-type inflammatory cytokines to skin explants and reconstructed epidermises, in which topically-applied IMD-006 normalized keratinocyte proliferation and differentiation. In 2D keratinocyte cultures, IMD-006 also decreased proliferation whilst promoting differentiation. It was internalized by keratinocytes and distributed to mitochondria. After treatment with IMD-006, changes to the morphology of the mitochondrial network, increases in mitochondrial ROS levels, and co-localization of mitochondria with lysosomes suggest it promotes mitochondrial degradation, a key step in keratinocyte differentiation. Therefore, our results show that IMD-006 could be a promising candidate for the topical treatment of psoriasis.

## Introduction

Psoriasis is a chronic, inflammatory skin disease that affects around over 100 million people worldwide. This debilitating disease presents as red, raised, scaly plaques on the skin that cause itching, burning and pain that greatly affect the quality of life of patients [1]. The most common form of psoriasis, and the one studied here, is Psoriasis Vulgaris (hereafter referred to as “psoriasis”). Currently, there is no curative treatment for psoriasis [2], and conventional treatments that aim to reduce symptoms can present low efficiency, such as topical anti-inflammatory agents, or have severe side effects and/or are highly expensive, such as systemic treatments with synthetic molecules or biologic immunomodulators. So, there is an unmet need to develop new drugs that could provide sustainable therapeutic effects.

The chronic inflammation found in the skin of psoriatic patients, depends on the activation and interaction of keratinocytes (KC) and infiltrating inflammatory immune cells. Indeed, in response to different factors (wounds, infection, stress, genetic predispositions etc.), KC release fragments of DNA linked to the antimicrobial peptide LL37 [1,3] that activate dendritic cells (DC), in turn leading to the activation and recruitment of T helper (Th)1 and Th17 cells, then polynuclear neutrophils and macrophages. The recruited immune cells produce pro-inflammatory cytokines that then act on KC, inducing hyperproliferation and abnormal differentiation of these cells [1]. Indeed, in healthy skin, a fine balance exists in which KC progress from a proliferating state in the stratum basale towards a fully differentiated state in the stratum corneum. During this differentiation process KC undergo a programmed cell death during which their organelles and nucleus are degraded and their plasma membrane is replaced by a lipids-proteins cross-linked structure called the cornified envelope [2,3]. In psoriatic lesions, KC proliferation increases whilst differentiation and cell death decrease, resulting in a thickening of the epidermis (acanthosis) and the stratum corneum (hyperkeratosis), the presence of nuclei in the stratum corneum (parakeratosis) and intercellular oedema (spongiosis) [1,3]. Thus, therapeutic strategies that are able to rebalance KC proliferation and differentiation may be beneficial for the treatment of psoriasis.

**Fig 1:**
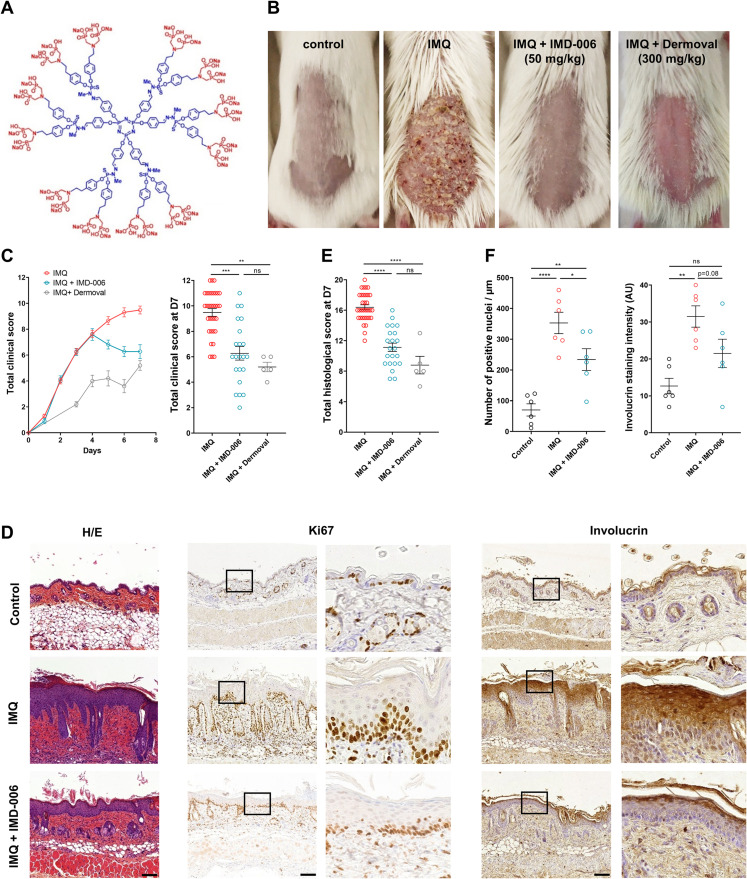
IMD-006 improves IMQ-induced psoriasis-like lesions in mice. **(A)** Two-dimensional structure of IMD-006. The cyclotriphosphazene core (N_3_P_3_) holding 6 phenoxymethyl-methylhydrazone branches is in blue and the 12 azabisphosphonate surface groups are in red. **(B)** Photographs of mouse skin taken at day 7. **(C)** Clinical scores were monitored daily by evaluating redness (erythema), scaling, and thickness of skin on a scale from 0 (none or normal) to 4 (severe). The total clinical score is the sum of these scores. The left panel shows evolution of clinical score over time and the right panel shows the final clinical score at day 7 of all animals from all groups. N = 33 for untreated mice, N = 23 for mice treated with IMD-006 (50 mg/kg) and N = 5 for mice treated with Dermoval (300 mg/kg). **(D)** Biopsies of the skin of control, imiquimod (IMQ), and treated (IMQ + IMD-006) mice were taken and fixed at day 7. The left panels show hematoxylin/eosin (H/E) staining, the central panels show IHC staining targeting the nuclear proliferation marker Ki67, and the right panels show IHC staining targeting the differentiation marker involucrin on representative skin sections. Scale bar 50 µm. **(E)** Histological score was assessed on the H/E stainings by evaluating acanthosis, hyperkeratosis, parakeratosis, spongiosis, and immune cell infiltrate on a scale of 0 (normal) to 4 (severe). Total histological score is the sum of these scores. N = 33 for untreated mice, N = 23 for mice treated with IMD-006 (50 mg/kg) and N = 5 for mice treated with Dermoval (300 mg/kg). **(F)** Immunodetection of Ki67 (left) and involucrin (right) was quantified. Statistical significance of differences was assessed using a One-way ANOVA with Tukey’s post test (for graphs in panels E and F) or a Kruskall-Wallis test with Dunn’s post test (for graph in panel **C)**.

Dendrimers are hyperbranched, multivalent, and polyfunctional nanodevices that display several advantages for biomedical applications [4], including the optimized targeting of drugs to relevant layers of the skin [5]. Dendrimers are synthesized in a stepwise fashion. Generally, low generation dendrimers have a perfectly defined structure. They are a promising alternative to poorly defined nanoparticles, such as polymeric or metallic ones, for biomedical development. Dendrimers are designed from a central core to which are linked one or several series of branched monomers. Each monomer is ended by a branching point which enables the dendritic growth of the molecule by addition of the next generation of branched monomers, leading to a “tree-like” structure. The synthesis ends with the addition of functional groups on the last-added series of branched monomers. The total number of series of branched layers determines the generation of the dendrimer [4]. A few types of dendrimers have shown anti-inflammatory properties per se in chronic and acute inflammatory animal models [6]. IMD-006, previously known as ABP dendrimer, is a generation 1 phosphorus-based dendrimer capped with 12 azabisphosphonate groups (Fig 1A), its molecular weight is 5817 Da, and its hydrodynamic radius is 2–3 nm. IMD-006 has strong immuno-modulatory and anti-inflammatory effects [7]. Indeed, it has been shown to protect mice against the development of several inflammatory diseases such as Rheumatoid Arthritis or Multiple Sclerosis [8–11]. In these models we have shown that the therapeutic efficacy of IMD-006 is mediated through its effects towards immune cells such as monocytes/macrophages, CD4^+^ T lymphocytes, and DC. Moreover, these immune-modulatory effects have been validated *ex vivo* on human immune cells [12–16]. In a pilot study, we have also shown that the topical application of IMD-006 has a therapeutic effect in the mouse model of imiquimod (IMQ)-induced psoriasis [17]. We have demonstrated that the therapeutic effect of the IMD-006 dendrimer is, at least in part, due to a decrease in the immune cell infiltrate, particularly macrophages, in the skin of treated mice. However, we show here that IMD-006 also acts on the other cell component that plays a pivotal role in the development of psoriasis, KC. Indeed, topical treatment with IMD-006 significantly reduced histopathological changes associated with KC dysfunction in the epidermis in murine and human models of psoriasis. We further showed that this was due to a direct effect on these cells, in particular IMD-006 decreased KC proliferation whilst promoting differentiation in 2D cultures. Moreover, IMD-006 was shown to target KC mitochondria, leading to lysosomal degradation of these organelles, a key step in KC differentiation [18,19]. This study, in conjunction with our previous work, suggests that IMD-006 may be a good candidate for the topical treatment of psoriasis.

## Results

### IMD-006 improves epidermal morphology by normalizing KC proliferation and differentiation, in both mouse and human psoriasis models

In similar experimental settings as previously used (S1 Fig) [17] we confirmed that the topical application of the IMD-006 dendrimer (Fig 1A) at 50 mg/kg in water improves the visual aspect of the skin of IMQ-induced psoriatic mice (Fig 1B). In these experiments 0.05% Dermoval, a cream based on the clobetasol propionate corticosteroid that is indicated for the treatment of moderate to severe plaque psoriasis, was used as gold standard at 300 mg/kg. The total clinical score of mice based on erythema (*i.e.*, redness), scaling, and thickness was significantly decreased with both IMD-006 and Dermoval (Fig 1C). Biopsies were taken at day 7 and histological features of the skins were assessed after hematoxylin/eosin (H/E) staining (Fig 1D). Quantification of acanthosis, hyperkeratosis, parakeratosis, spongiosis, and immune cell infiltrate (including polynuclear neutrophil (PNN) abscesses, so-called Munroe’s abscesses) (S1 Fig) showed a significant decrease of the total histological score with both IMD-006 and Dermoval (Fig 1E). Immunohistochemical (IHC) stainings for Ki67 and involucrin were also performed on skin biopsies taken at day 7. Ki67 is a nuclear protein that is a marker of cell proliferation. As expected, its expression was strongly increased in IMQ-diseased mice (Fig 1D) and the number of Ki67 positive nuclei was significantly decreased in IMD-006-treated mice when compared to untreated mice (Fig 1F). Involucrin is an early marker of the differentiation process of KC. In normal skin, it is expressed only in the upper spinous and the granular layers, but in psoriatic lesions its level is increased as it is expressed earlier, from the first suprabasal layer. The same was observed in IMQ-treated mice (Fig 1D). In IMD-006-treated animals, the expression pattern of involucrin was normalized (Fig 1F), with expression found mainly in the upper layers of the epidermis (Fig 1D). Furthermore, a decrease of the infiltration of immune cells was revealed by the H/E staining in skin of IMD-006-treated mice (Fig 1D). This was in align with the well-known immunomodulatory and anti-inflammatory properties of the molecule [12]. Therefore, we quantified the infiltration of relevant immune cells in the skin by IHC. This showed a normalization of CD3-positive cells (*i.e.*, T lymphocytes) and F4/80-positive cells (*i.e.*, mainly macrophages), and a significant decrease of Ly6C-positive cells (*i.e.*, PNN) (S2 Fig).

**Fig 2:**
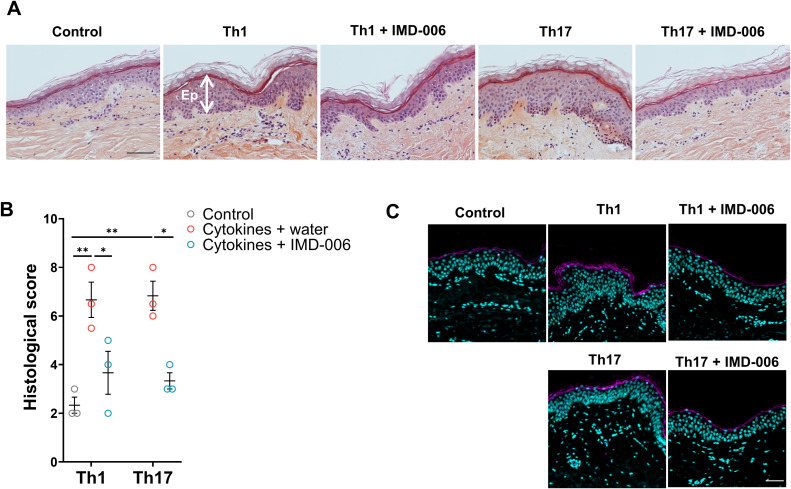
IMD-006 improves both Th1- and Th17-induced psoriasis-like alterations of cultured normal healthy human skin explants. **(A)** H/E staining of representative sections of skin explants. Scale bar = 50 µm. Ep, epidermis. **(B)** Histological score was assessed on the H/E stainings by evaluating acanthosis, hyperkeratosis, parakeratosis, spongiosis, and immune cell infiltrate on a scale of 0 (normal) to 4 (severe). Total histological score is the sum of these scores, N = 3. Statistical significance of differences was assessed using a Kruskall-Wallis test with Dunn’s post test. **(C)** IHC staining targeting involucrin (in magenta) with DAPI nucleic counterstain (in cyan) on representative skin sections. Scale bar = 50 µm.

In addition to the IMQ-induced psoriatic mouse model, we also implemented *ex vivo* human full thickness models of psoriasis induced by the action of either Th1-type (interleukin (IL)-6, TNF, and IL-1α at 5 ng/mL) or Th17-type (IL-17 and IL-22 at 5 ng/mL) cytokines added in the culture medium of normal healthy skin explants. The effect of IMD-006 was assayed by its topical application concomitantly with the addition of the pathogenic cytokines in the culture medium of explants. The H/E staining of the skin sections showed a marked reduction of the epidermal thickness when IMD-006 was applied, in both Th1 and Th17 models (Fig 2A). Based on the same criteria as the IMQ-induced mouse model, the total histological scores of the epidermis of the skins treated with IMD-006 was significantly decreased in both cytokine-induced models (Fig 2B). Finally, the IHC staining for involucrin also shows a normalization of this marker upon treatment with IMD-006 in both *ex vivo* Th1 and Th17 human models (Fig 2C), thereby confirming the therapeutic efficacy of IMD-006 dendrimer in mouse and human models of psoriasis.

### IMD-006 decreases proliferation and promotes differentiation of human KC *in vitro*

**Fig 3:**
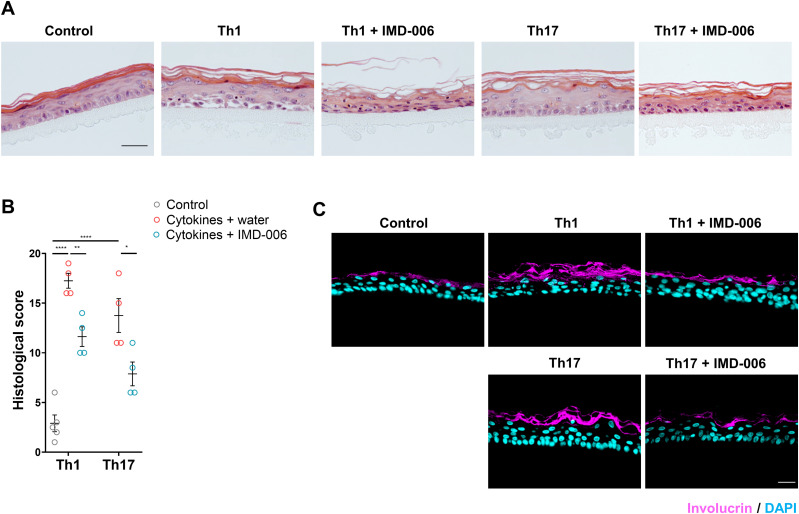
IMD-006 improves both Th1- and Th17-induced psoriasis-like alterations of N/TERT-1 cell-based 3D reconstructed human epidermis. **(A)** H/E staining of representative sections of reconstructed human epidermis (RHE). Scale bar = 50 µm. **(B)** Histological score was assessed on the H/E stainings by evaluating acanthosis, hyperkeratosis, parakeratosis, spongiosis, stratum corneum thickness, and the presence of stratum granulosum on a scale from 0 (normal) to 4 (severe). The total histological score is the sum of these scores, N = 4. Statistical significance of differences was assessed using a one-way ANOVA with Tukey’s post-test. **(C)** IHC staining targeting involucrin (in magenta) with DAPI nucleic counterstain (in cyan) on representative RHE sections. Scale bar = 40 µm.

In order to determine whether the effects of IMD-006 dendrimer on the epidermis could be due to a direct effect on KC, we used three-dimensional reconstructed human epidermis (RHE), in which the only cellular component is human KC of the N/TERT-1 cell line, hence without any immune cells. Once the RHE has been obtained, again either Th1-type or Th17-type cytokines were added to the culture medium to induce a psoriasis model. When topically applied to the RHE concomitantly with the addition of cytokines in the culture medium, IMD-006 also improved the epidermal thickness and morphology (Fig 3A), significantly decreased the total histological scores (Fig 3B), and normalized the expression of involucrin (Fig 3C). Therefore, IMD-006 has a direct effect on human KC.

**Fig 4:**
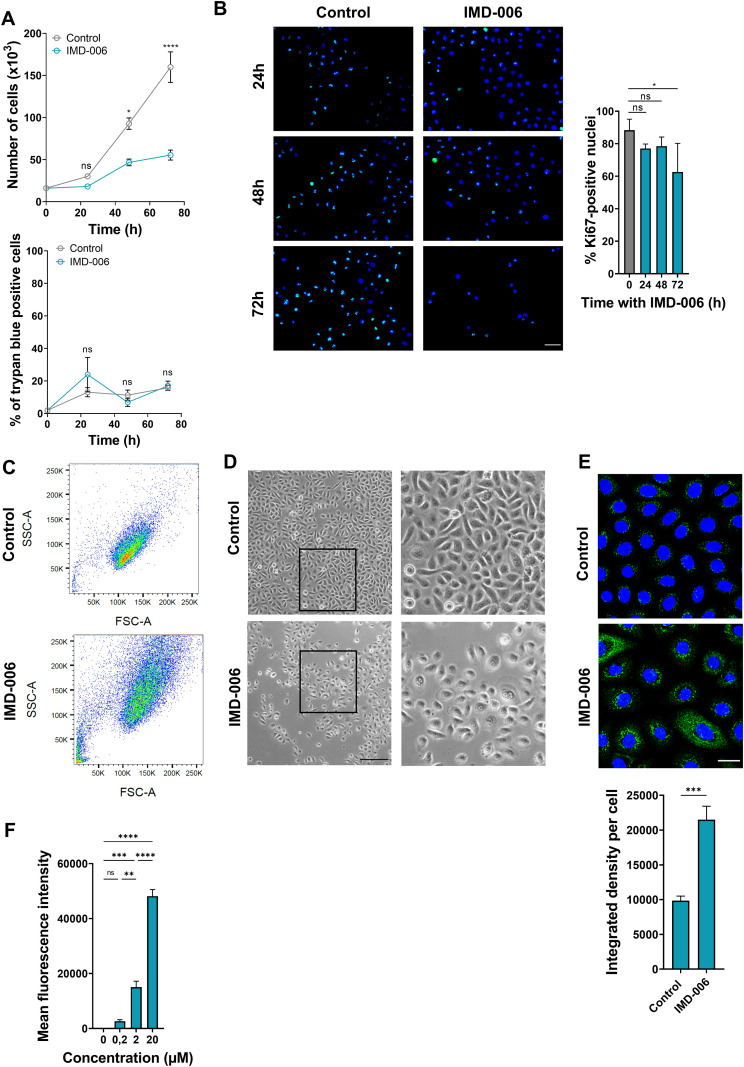
IMD-006 decreases proliferation and increases differentiation of N/TERT-1 KC *in vitro.* **(A)** N/TERT-1 KC were cultured with IMD-006 (20µM), or not (control), for 24, 48 and 72 h. At each time-point, cells were counted (top panel) and stained with Trypan blue to evaluate cell mortality (bottom panel). N = 3 independent experiments. **(B)** N/TERT-1 KC were cultured on glass coverslips with IMD-006 (20 µM), or not, for 24, 48 and 72 h. Cells were fixed and immunostaining targeting the proliferation marker Ki67 was performed with DAPI counterstaining. Representative images are shown (left panel) and the proportion of Ki67-positive nuclei was calculated in 4 independent experiments on at least 5 images per condition (right panel). **(C, D)** N/TERT-1 KC were cultured with IMD-006 (20 µM), or not, for 72 h. Cells were harvested to perform morphological assessment by flow cytometry (C; FSC-A, Forward Scatter, indicates the size of the cells; SSC-A, Side Scatter, indicates their granularity; one representative result shown), or bright field microscopy images of cells in culture were taken (D, one representative result shown, scale bar = 500µm). **(E)** N/TERT-1 KC were cultured with IMD-006 (20 µM), or not, for 72 h. Cells were fixed and immunostaining targeting the differentiation marker involucrin was performed with DAPI counterstaining. Representative images are shown (top panel) and the intensity of involucrin staining per cell was calculated in 5 images per condition (bottom panel). **(F)** N/TERT-1 KC were cultured for 1 h at 37 °C with IMD-006-NIR (fluorescent analog of IMD-006) at increasing concentrations (0.2, 2, 20 µM). Intracellular fluorescent of the cells was quantified by flow cytometry. Statistical significance of differences was assessed using Student’s t-test for comparison of 2 groups (A, E) or a one-way ANOVA with Tukey’s post-test for 3 groups or more **(B, F)**.

**Fig 5:**
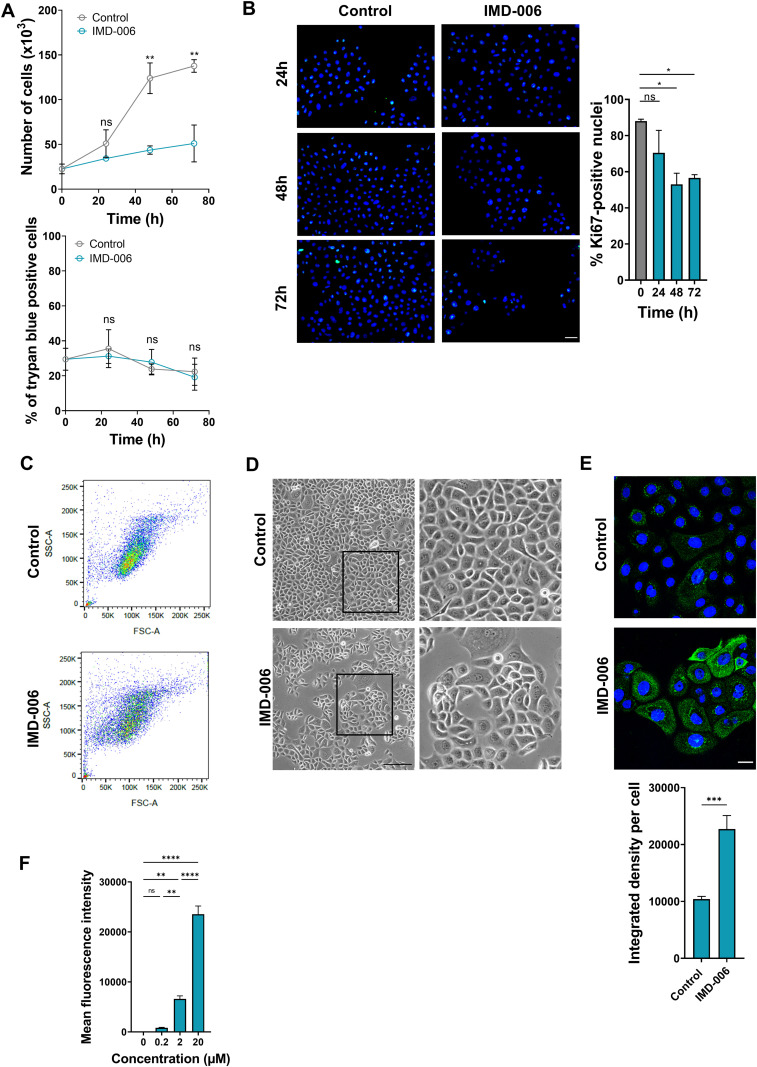
IMD-006 decreases proliferation and increases differentiation of human primary KC *in vitro.* **(A)** Primary KC from 3 donors were cultured with IMD-006 (20µM), or not (control), for 24, 48 and 72 h. At each time-point, cells were counted (top panel) and stained with Trypan blue to evaluate cell mortality (bottom panel). **(B)** Primary KC from 3 donors were cultured on glass coverslips with IMD-006 (20 µM), or not, for 24, 48 and 72 h. Cells were fixed and immunostaining targeting the proliferation marker Ki67 was performed with DAPI counterstaining. Representative images are shown (left panel) and the proportion of Ki67-positive nuclei was calculated for each donor on at least 5 images per condition (right panel). **(C, D)** Primary KC from 3 donors were cultured with IMD-006 (20 µM), or not, for 24, 48 and 72 h. Cells were harvested to perform morphological assessment by flow cytometry (C; FSC-A, Forward Scatter, indicates the size of the cells; SSC-A, Side Scatter, indicates their granularity; one representative result shown), or bright field microscopy images of cells in culture were taken (D, one representative result shown, scale bar = 500µm). **(E)** Primary KC from 3 donors were cultured with IMD-006 (20 µM), or not, for 72 h. Cells were fixed and immunostaining targeting the differentiation marker involucrin was performed with DAPI counterstaining. Representative images are shown (top panel) and the intensity of involucrin staining per cell was calculated in 5 images per condition (bottom panel). **(F)** Primary KC from 3 donors were cultured for 1 h at 37 °C with IMD-006-NIR (fluorescent analog of IMD-006) at increasing concentrations (0.2, 2, 20 µM). Intracellular fluorescent of the cells was quantified by flow cytometry. Statistical significance of differences was assessed using Student’s t-test for comparison of 2 groups (A, E) or a one-way ANOVA with Tukey’s post-test for 3 groups or more **(B, F)**.

The direct effect of IMD-006 on the proliferation and the differentiation of human KC was further demonstrated in monolayer cultures of both the N/TERT-1 cell line and human primary KC. Both KC were cultured in the presence of IMD-006 for 24, 48, and 72 h. Significant lower numbers of cells were counted after 48 and 72 h for both N/TERT-1 (Fig 4A, upper panel) and primary (Fig 5A, upper panel) KC. To rule out any toxicity of IMD-006, Trypan blue staining was performed showing the same percentages of Trypan blue positive cells at all the culture time points for both N/TERT-1 (Fig 4A, lower panel) and primary (Fig 5A, lower panel) KC. These results strongly suggest an anti-proliferative effect of IMD-006 on human KC. This was further confirmed by the Ki67 staining of KC that was significantly decreased after 72 h for N/TERT-1 KC (Fig 4B) and after 48 and 72 h for primary KC (Fig 5B), demonstrating thereby that IMD-006 decreases the proliferation of human KC *in vitro*.

Moreover, N/TERT-1 and primary KC exposed to IMD-006 for 72 h presented an increase in their size and granularity, as evidenced by flow cytometry (Figs 4C and 5C) and microscopy (Figs 4D and 5D), that is indicative of a differentiated state. Finally, in both N-TERT-1 and primary KC, IMD-006 significantly increased the expression of the differentiation marker involucrin (Figs 4E and 5E).

### After 1 h, IMD-006 is internalized in N/TERT-1 KC where it is distributed to mitochondria

**Fig 6:**
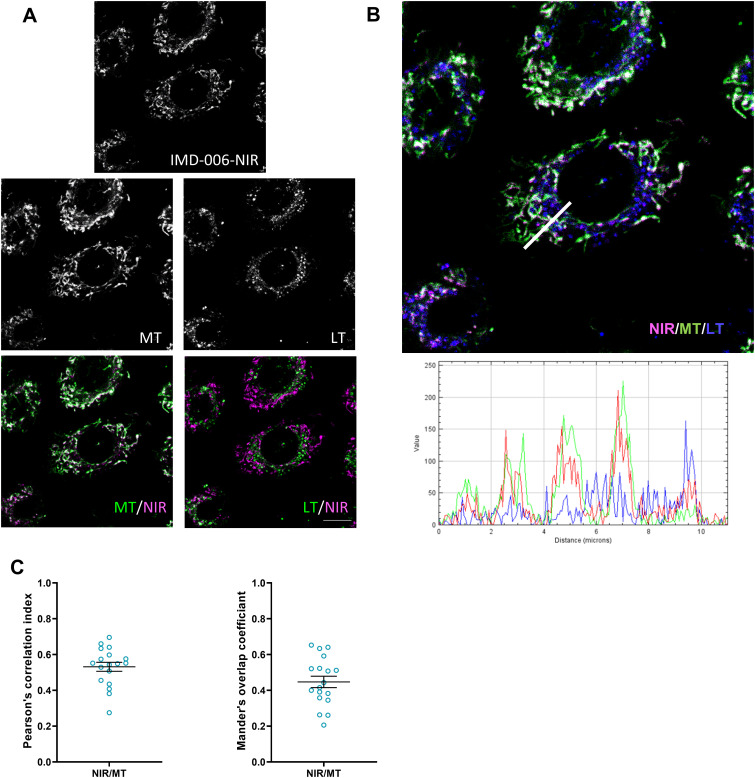
After 1 h, IMD-006 is distributed to mitochondria in N/TERT-1 KC, modifying their network morphology. **(A)** N/TERT-1 KC were cultured for 1 h with IMD-006-NIR (fluorescent analog of IMD-006) at 20 µM. Then, cells were stained with MitoTracker™ Green FM (MT, to visualize mitochondria) and Lysotracker Red DND-99 (LT, to visualize lysosomes), and observed by confocal microscopy. Scale bar = 10 µm. **(B)** The fluorescence profiles of each channel along a plot line (shown in white on the microscopy image) are shown below the image (created using the RGB profiler plug-in from Image **J)**. **(C)** On images from 3 independent experiments (6 images for each), colocalisation of IMD-006-NIR with mitochondria (MT) was assessed by calculating the Pearson’s correlation index and the Mander’s overlap coefficient using Image J software.

We then tried to decipher the mechanism of action of IMD-006 that leads to decreased proliferation and heightened differentiation of KC. We showed that a fluorescent analog of the dendrimer IMD-006 (IMD-006-NIR, emitting in the near infrared) [17] was internalized by both N/TERT-1 and primary KC in a dose dependent manner (Figs 4F and 5F). This was an active process since internalization was inhibited at 4°C (data not shown). We then determined the subcellular location of the molecule. This revealed that IMD-006-NIR rapidly co-localized with mitochondria within 1 h after its addition to the culture medium of N/TERT-1 (Figs 6A and 6B), with high Pearson’s and Mander’s co-localization coefficients (Fig 6C), whereas little IMD-006-NIR was found to co-localize with lysosomes at this early time point (Fig 6B).

### After 72 h, IMD-006 induces modifications of the mitochondrial network morphology in N/TERT-1 KC, and increases both mitochondrial ROS level and mitochondria content in lysosomal structures

**Fig 7:**
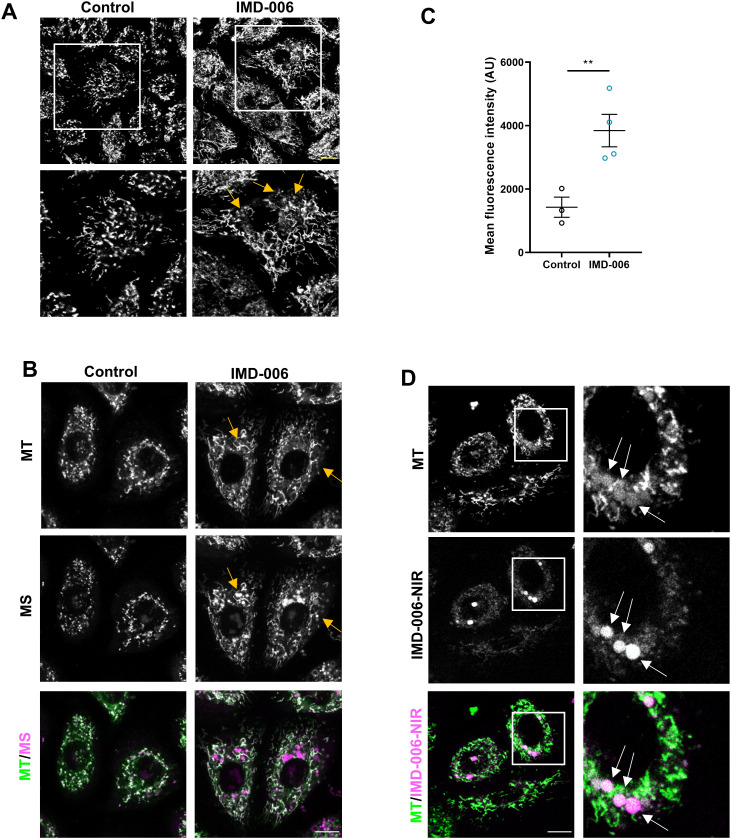
After 72 h, IMD-006 modifies the mitochondrial network and increases mitochondrial ROS in N/TERT-1 KC. **(A)** N/TERT-1 KC were cultured for 72 h with IMD-006 (20µM), or not (control). Then, cells were stained with MitoTracker™ Green FM (MT, to visualize mitochondria) before observation by confocal microscopy. Yellow arrows show small, fragmented mitochondria in the perinuclear region. Scale bar = 10 µm. **(B)** N/TERT-1 KC were cultured for 72 h with IMD-006 (20µM), or not. Then, cells were stained with MitoTracker™ Green FM (MT, to visualize mitochondria) and MitoSOX™ red (MS, to assess mitochondrial superoxide levels) before observation by confocal microscopy. Yellow arrows show small, fragmented mitochondria in the perinuclear region. Scale bar = 10 µm. **(C)** N/TERT-1 KC were cultured 72 h with IMD-006 (20 µM), or not. Then, cells were stained with MitoSOX™ red only and analyzed by flow cytometry to quantify mitochondrial superoxide levels. Statistical significance of differences was assessed using Student’s t-test. **(D)** N/TERT-1 KC were cultured for 72 h with IMD-006 and its fluorescent analog (IMD-006-NIR) in a ratio of 9:1 (total 20 µM). Then, cells were stained with MitoTracker™ Green FM (MT, to visualize mitochondria) before observation by confocal microscopy. White arrows show small, fragmented mitochondria and colocation thereof with IMD-006-NIR. Scale bar = 10 µm.

After 72 h of culture with a mix of IMD-006 with 10% of its IMLD-006-NIR analog, time point at which we observed a strong effect of the dendrimer on the proliferation and differentiation of KC, mitochondrial morphology was greatly modified. Indeed, small, fragmented mitochondria accumulated in the perinuclear region of KC treated with IMD-006 (Fig 7A), consistent with an increased mitochondrial fission. Interestingly, previous studies have shown that fragmented mitochondria often present high levels of reactive oxygen species (ROS) that can serve as a signal for lysosomal clearing of these dysfunctional organelles [19]. Consistent with the hypothesis that IMD-006 induces such a process in KC, we found that mitochondrial ROS levels were increased in treated cells and particularly within the fragmented, perinuclear mitochondria (Figs 7B and 7C). Importantly, IMD-006-NIR was also located within these dysfunctional organelles (Fig 7D).

**Fig 8:**
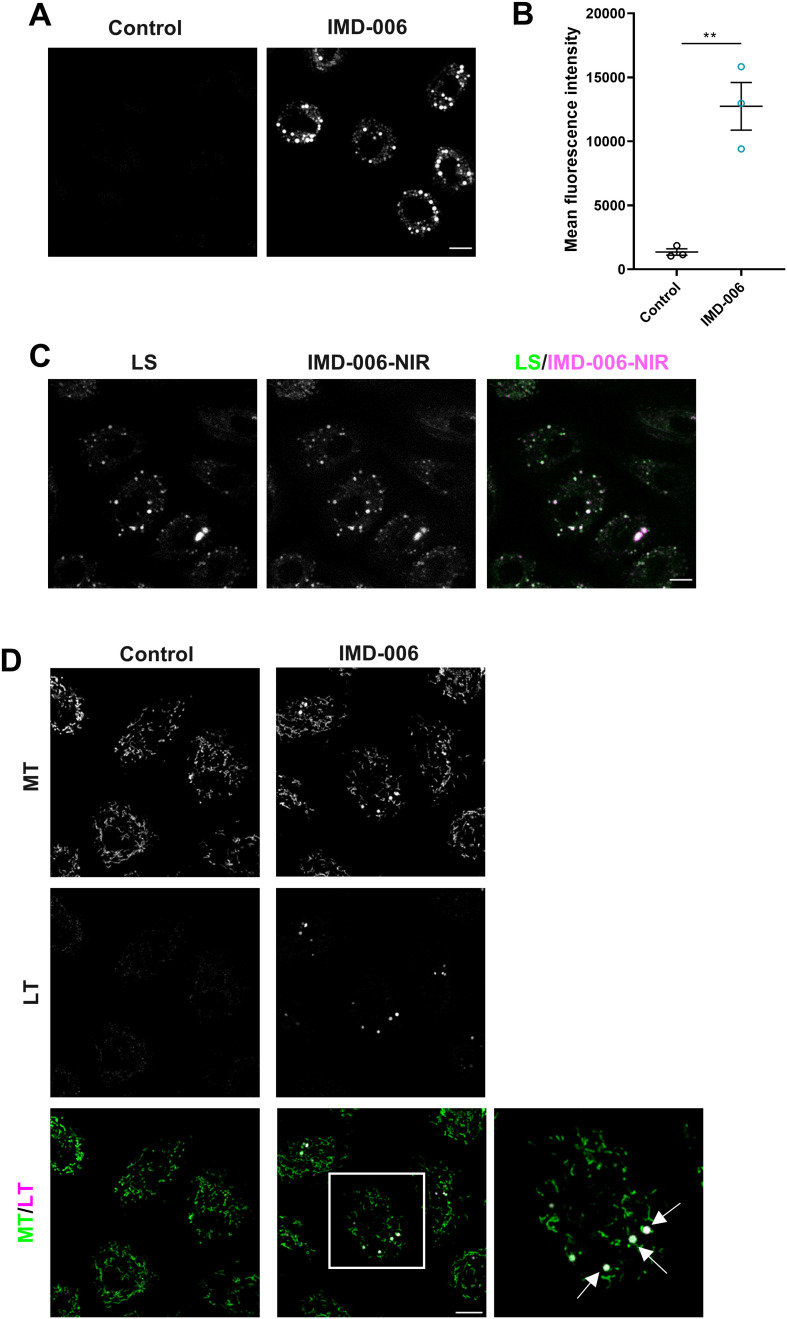
After 72 h, IMD-006 increases lysosomes and their colocation with mitochondria in N/TERT-1 KC. **(A, B)** N/TERT-1 KC were cultured for 72 h with IMD-006 (20 µM), or not (control). Then, cells were stained with Lysosensor Green DND-26 (to visualize lysosomes) before observation by confocal microscopy (A, scale bar = 10 µm), and quantification by flow cytometry **(B)**. Statistical significance of differences was assessed using Student’s t-test. **(C)** N/TERT-1 KC were cultured for 72 h with IMD-006 and its fluorescent analog (IMD-006-NIR) in a ratio of 9:1 (total 20 µM). Then, cells were stained with Lysosensor Green DND-26 (LS, to visualize lysosomes) before observation by confocal microscopy (scale bar = 10 µm). **(D)** N/TERT-1 KC were cultured for 72 h with IMD-006 (20 µM), or not. Then, cells were stained with MitoTracker™ Green FM (MT, to visualize mitochondria) and with Lysotracker Red DND-99 (LT, to visualize lysosomes) before observation by confocal microscopy. White arrows show colocation of mitochondria and lysosomes. Scale bar = 10 µm.

We next analyzed the effect of IMD-006 on lysosomes in KC treated with IMD-006. After 72 h of treatment, IMD-006 significantly increased the content of lysosomes in KC (Figs 8A and 8B). Moreover, IMD-006-NIR co-localized with lysosomes at this time point (Fig 8C) and, as stated above, we had also found that IMD-006-NIR located within the small, fragmented perinuclear mitochondria after 72 h (Fig 7D). Thus, these results strongly suggest that the presence of the dendrimer within mitochondria leads to their subsequent degradation by lysosomes. Accordingly, using video microscopy we observed that in KC treated with a mix of IMD-006 and IMD-006-NIR, the mitochondrial network was modified over time with the appearance of small, fragmented mitochondria that co-localize with lysosomes before rapidly disappearing, which is consistent with mitochondrial clearing by lysosomes (Supplementary S2 Video, Supplementary S1 and S3 Videos being controls thereof).

## Discussion

IMD-006 dendrimer is known to display immuno-modulatory and anti-inflammatory effects towards various human immune cells, including monocytes [13], T lymphocytes [14], NK cells [15], and DC [16], and induces expression of the nti-inflammatory cytokine IL-10 [12]. This led us to propose the dendrimer as a therapeutic molecule to treat various inflammatory diseases. We previously demonstrated the potential of IMD-006 in mouse models of Rheumatoid Arthritis [8,11] and Multiple Sclerosis [10], and more recently of psoriasis [17]. Here, we confirmed that IMD-006 exhibits great potential against psoriasiform dermatitis induced by IMQ in mice, and showed for the first time that IMD-006 could also target KC. Since changes in these epithelial cells is a hallmark of psoriasis pathogenesis, this is a novel argument to propose IMD-006 as a treatment for this disease.

Psoriasis, a chronic inflammatory skin disease, affects approximately 3% of the population. It is currently considered as a systemic disorder with a predominant skin involvement. Its pathogenesis involves over-activation of cells of both innate and acquired immunity, and hyperproliferation and abnormal differentiation of KC leading to impairment of the epidermal barrier function and retention of nuclei in the stratum corneum [1]. Indeed, the uppermost KC layers in the epidermis act as vital multiple barriers between the organism and its environment through their involvement in innate immunity, their high mechanical strength, their ability to detoxify ROS, to limit water loss, to reduce the penetration of UV radiation, and to prevent the infiltration of allergens and microorganisms. The ultimate steps in KC terminal differentiation result in the degradation and clearing of all cellular organelles, including the nucleus and mitochondria, likely through autophagy processes.

Our study confirmed the potential anti-psoriatic action of topically applied IMD-006 using an *ex vivo* human psoriasis model generated by the action of T helper (Th)1-type and Th17-type inflammatory psoriasis-characteristic cytokines on skin explants in culture. Importantly, IMD-006 was also shown to partly reverse the alterations in KC proliferation/differentiation balance and morphological impairments in 3D reconstructed epidermises treated with the same cytokine cocktails. Our results showed that IMD-006 is internalized by KC in 2D cultures of both the N/TERT-1 cell line and primary epidermal cells, then is rapidly addressed to mitochondria. Subsequent fission of these organelles was observed, together with a reduced KC proliferation and signs of differentiation. Changes to the morphology of the mitochondrial network, increases in mitochondrial ROS levels, and the addressing of mitochondria to lysosomes all point to the induction of mitochondria degradation by the IMD-006 dendrimer. The dendrimer has also been shown to inhibit proliferation of conventional CD4+ T lymphocytes [14]. In oppose to these findings, IMD-006 has previously been shown to promote the growth of γδ T cells and natural killer cells [15]. Whether these data could be explained by different mitochondrial response remains to be tested.

Importantly, it has been shown that such processes are crucial steps in KC differentiation, and therefore inducing them in psoriatic lesions should improve the deregulated proliferation/differentiation balance in KC. Indeed, mitochondrial depolarization, fragmentation and clearing in an acidic cellular compartment, probably autophagolysosomes, in the upper KC layers are necessary to and drive epidermal differentiation by the NIX-DRP1 pathway [18]. Inhibition of either autophagy or lysosome activity in organotypic human skin was shown to block KC differentiation [19]. Moreover, the high calcium ion concentration that promotes the expression of KC differentiation markers, also triggers mitochondrial defects and cytosolic release of ROS, thus promoting mitophagy [19]. Release of ROS is also necessary for KC differentiation through notch signaling [20,21]. What is more, it is increasingly evident that autophagy is impaired in psoriasis due to lysosomal dysfunction and/or mTOR (a central regulator of cellular metabolism) activation, and this contributes to the pathogenesis [22–24]. For example, long-term exposure of KC to either TNF or IL-17, two key inflammatory cytokines in psoriasis, reduces autophagy [22].

On the basis of these data, we speculate that the mitochondria fission and translocation to acidic compartments, either lysosomes or autophagolysosomes, following mitochondria-IMD-006 interaction induces KC differentiation together with a reduction in KC proliferation, as we observed *in vitro*, and may regulate the abnormal KC differentiation and proliferation in IMQ-related psoriasiform skin lesions in mice. In line with this hypothesis, the number of mitochondria is higher in KC of lesional psoriatic skin compared with normal skin [25], the expression of Dynamin-related protein 1 (Drp1), a mitochondrial fission factor, is reduced at the mRNA level in the lesional skin of psoriasis patients [26]. mTOR inhibition in the IMQ psoriasis model alleviates clinical signs and restores KC differentiation [24]. IMQ is known to interact with mitochondrial fusion/fission cycle, and this is used to treat skin carcinoma, the cancer cells being far more sensitive than the normal ones [27].

A similar mode of action has been proposed for the antipsoriatic drug dithranol, also called anthralin that targets KC and their cross-talk with neutrophils [28], *i.e.*, accumulation in KC mitochondria and disruption of the mitochondrial membrane potential [29]. Moreover, perinuclear arrangements of altered and vacuolated mitochondria has been observed using transmission electron microscopy, only in KC [30]. This is reminiscent of our observation (Fig 6B).

An alternative or complementary explanation for the effect of the dendrimer on cell proliferation is the following. The elevated turnover rate of KC in psoriatic skin requires enhanced oxidative phosphorylation, which takes place inside mitochondria, in order to sustain the increased demand of ATP. Therefore, the observed degradation of mitochondria would reduce the production of energy for the KC and therefore their proliferation.

In conclusion, we have identified human KC as a new therapeutic target of the immuno-modulatory and anti-inflammatory IMD-006 dendrimer. Together with its effects on immune cells, the combined properties of the molecule on the proliferation/differentiation balance of KC underpin its anti-psoriatic effect. IMD-006 has already been evaluated regarding its early safety and biodistribution in different species [31–33]. It is the hit of a promising new class of anti-inflammatory compounds, especially to improve psoriasis. However, its therapeutic efficacy to improve this disease should be enhanced by formulating it in appropriate nanosystems designed for epidermal permeation and delivery [34].

## Materials and methods

### Synthesis of the IMD-006 and IMD-006-NIR dendrimers

The IMD-006 dendrimer and its near infra-red (NIR) fluorescent analogue IMD-006-NIR were synthesized at the “Laboratoire de Chimie de Coordination” (Toulouse, France). Their synthesis has been previously described [17,35].

### Imiquimod-induced mouse model of psoriasis

8-week old female BALB/c mice (Envigo, France) were housed in groups of 5 in plastic cages in a controlled environment (temperature of 22 ± 2 °C and relative humidity of 50 ± 15%) with 12 h cycles of light and darkness. Food and drinking water were provided ad libitum. Mice were acclimatized for a week before starting the experiment. First, fur was removed from the back skin of mice by shaving then applying depilatory cream on a surface area of approximately 1.5 x 2.5 cm. 24 h later, and then every afternoon for 7 consecutive days, 80 mg of a cream containing 5% IMQ (Aldara, Meda), or a 2% wt Xanthan-based hydrogel (Aroma Zone) used as a control, was applied to this area and massaged into skin. Every morning, the skin was cleaned with a wet cotton pad then 100 µL of water or IMD-006 (50 mg/kg) was applied and massaged into skin. Each afternoon, before applying IMQ or Xan, a clinical score was performed based on three criteria: redness, scaling, and thickness. Each criterion was scored on a scale of 0–4: 0 = normal, 1 = slight, 2 = moderate, 3 = severe, and 4 = very severe. The sum of the individual scores gave the total score. All experimental procedures involving animals were performed according to the French and European guidelines and laws. All experiments were conducted by authorized investigators in accordance with the ARRIVE guidelines and were approved by the government-accredited animal care and use committee (US006/ CREFRE Ethical Committee, Toulouse, France). Mice were monitored twice daily to ensure their wellbeing. At the time of sacrifice, animals were anesthetized with isoflurane prior to cervical dislocation to ensure the absence of suffering.

### KC culture

N/TERT-1 KC [36] were kindly provided by J. Rheinwald (Harvard Medical School, Boston, USA) with the help of R. Debret (the Tissue Biology and Therapeutic Engineering Laboratory, Lyon, France). Primary KC were isolated from human skin samples from foreskin surgery on newborn children (obtained from Hôpital des Enfants CHU Purpan, Toulouse, France, with parental permission and in accordance with ethical guidelines and French law on bioethics). N/TERT1 and primary KC were cultured in Dermalife medium (Lifeline Cell Technology) with 1% penicillin/streptomycin (P/S, Sigma Aldrich) at 37°C in a humid atmosphere with 5% CO_2_. Cells were regularly passaged when they reached between 70 and 80% confluence (with a maximum of 3 passages for primary cells). Images of cells in culture were acquired with an EVOS M5000 microscope.

### Production of reconstructed human epidermises

N/TERT-1 KC were harvested and resuspended in EpiLife medium (Life Technologies) supplemented with 1.5 mM of calcium (Sigma Aldrich), 1% of HKGS (Human Keratinocyte Growth Supplement, Life Technologies) and 1% P/S. Then, 3.5x10^5^ cells were seeded in culture inserts (Millicell, 12 mm in diameter, polycarbonate, 0.4 µm pores; Millipore) placed in 6-well culture plates (Falcon) containing 2.5 mL of supplemented EpiLife medium and cultured for 48 h until a confluent layer of cells was obtained. Then, cultures were placed at the air-liquid interface by decreasing the volume of medium below the insert to 1.5 mL, which was supplemented with vitamin C (50 μg/mL, Life Technologies) and in KGF (Keratinocyte Growth Factor, 10 ng/mL, Sigma Aldrich). Over the next 12 days, the culture inserts were incubated at 37°C with 50% relative humidity and the culture medium was renewed every two days. Cytokine and dendrimer treatments ran from day 12–14 (48 h).

### Culture of human skin explants

All experimental procedures involving human samples were performed according to the French and European guidelines and laws. Samples of female abdominal skin were provided by Genoskin (Toulouse, France) following written informed consent of the donors, and the approval of the French Ministry of Research (#AC-2017-2897). 10 mm punch biopsies of the skin samples were placed in the same 12 mm culture inserts used for RHE production in 6-well culture plates. Biopsies were cultured so as the surface of the skin was at the air-liquid interface in a 1:1 ratio of DMEM (Dulbecco’s Modified Eagle Medium, Gibco) supplemented with 10% fetal calf serum (FCS, Gibco) and Epilife supplemented with vitamin C (50 μg/mL) and in KGF (10 ng/mL).

### Induction of a psoriatic phenotype in human skin explants and RHE and treatment with IMD-006

Human skin explants and RHE were treated or not with pro-inflammatory cytokines (Peprotech) to induce psoriasis-like phenotypes for 48 h. Th1 type cytokines (TNF, IL-6 and IL-1A) or Th17 type cytokines (IL-17 and IL-22) were added to cell culture medium at 5 ng/mL each. Concomitantly, human skin explants and RHE were topically treated or not with the IMD-006 dendrimer (diluted in ultrapure water to 100 µM from a stock solution at 2 mM). For this, 100 µL of IMD-006 (or ultrapure water for controls) was deposited on the surface of the explants or RHE.

### Treatment of KC monolayer cultures with IMD-006/IMD-006-NIR

IMD-006 or IMD-006-NIR was diluted in the culture medium of KC to the indicated concentrations from a stock solution at 2 mM prepared in ultrapure water and stored at −20 °C.

### Histopathology

At the end of the experiments, the back skin of mice, the punches of human skin or the RHE were collected and fixed in 4% formalin for 24 h. Then, they were embedded in paraffin, sliced (5 µm) and deposited on slides for hematoxylin/eosin (H/E) staining (performed by the CREFRE (Inserm UMS006) histopathology platform, Toulouse). Images were obtained with a Panoramic Slide Scanner, scanned at 40X magnification. Histological analysis and scoring of the slides were performed based on the following criteria for mouse and human skin explants: acanthosis, hyperkeratosis, parakeratosis, spongiosis and immune cell infiltration. For RHE, the following criteria were used: epidermal thickness, spongiosis, parakeratosis, hyperkeratosis, cohesion of the stratum corneum, and the presence of the stratum granulosum. Each criterion was scored on a 0–4 scale: 0 = normal, 1 = slight, 2 = moderate, 3 = severe, and 4 = very severe. The sum of the individual scores gave the total score.

### IHC of mouse skin samples

Slices obtained from the same paraffin blocks as those used for H/E staining were used to perform IHC analysis of the skin to study Ki67 and involucrin expression. After heating to dewax slides, sections were rehydrated in successive baths of Xylene, then ethanol before placing them in water. For Ki67, CD3 and Ly6C staining, antigen retrieval was performed in citrate buffer (pH 6, at 95°C for 20 min). For F4/80, antigen retrieval was performed using proteinase K antigen retrieval solution (Abcam). Endogenous peroxydase-blocking solution (Dako REAL) was applied for 10 min, Background Buster (Innovex) was used to block endogenous biotin for 20 min and non-specific antibody binding sites were blocked with a phosphate buffer saline (PBS) containing 3% bovine serum albumin (BSA, Gibco) and 2.5% goat serum. Sections were incubated with Ki67 (Abcam, reference ab16667 diluted at 1/200), involucrin (Covance, reference PRB-140C diluted at 1/500), CD3 (Abcam, ab16669 diluted at 1/100), Ly6C (Santa Cruz Biotechnology, 6A608 diluted at 1/100) or F4/80 (BioRad, MCA497R diluted at 1/100) primary antibodies diluted in PBS with 3% BSA for 1 h at room temperature. Then, sections washed and incubated with a goat anti-rabbit biotinylated secondary antibody (Vector, reference BP-9100-50 diluted at 1/250) or a goat anti-rat (BioRad, STAR131B diluted at 1/50) for 30 min at room temperature, followed by incubation with avidin/horseradish peroxidase (HRP) complex (Vector Laboratories) for 30 min. To reveal staining, 3,3’-Diaminobenzidine (Vector) was applied to sections for 5 min. Finally, nuclei were counterstained with hematoxylin for 3 min. After staining, sections were dehydrated in ethanol 100% then xylene before mounting. Images were obtained with a Pannoramic Slide Scanner (3DHistech) at 40X magnification.

### Mouse IHC quantification

Quantification of IHC stainings was performed on the previously scanned sections. On each skin section, 3 representative epidermal areas were delimited on the program CaseViewer. The percentage of involucrin-positive pixels per µm^2^ was quantified with the DensitoQuant feature of the Quantcenter plug-in and Ki67 positive nuclei per µm^2^ were counted manually.

### IHC of human skin explants and RHE

Slices obtained from the same paraffin blocks as those used for H/E staining were used to study involucrin expression. After heating to dewax slides, sections were rehydrated in successive baths of Xylene, then ethanol before placing them in water. Antigen retrieval was performed by placing the slides in a glycine buffer (50 mM in water, then adjusted to pH 3.5) for 30 min at 95°C. Saturation was achieved using PBS with 0.05% Tween (Sigma Aldrich) and 3% FCS for 1 h, before incubation with the anti-involucrin primary antibody (Covance, reference PRB-140C diluted at 1/1000 in PBS with 0.05% Tween and 3% BSA) for 1 h at room temperature. Then, after washing, sections were incubated with the goat anti-rabbit secondary antibody (ThermoFisher, reference A-11008, diluted at 1/1000 in PBS with with 0.05% Tween and 3% BSA) for 1 h before counterstaining with 4’,6-diamidino-2-phenylindole (DAPI, ThermoFisher, 1 µM, 10 min). Finally, the slides were washed 3 times and mounted. Images were acquired with a Nikon 90i optical microscope with NIS-Element software.

### Cell counting and viability studies

N/TERT-1 or primary human KC were seeded in 24-well plates (2 × 10^4^ cells/well) and 24 h later were treated or not with IMD-006. Every 24 h, after staining with 10% Trypan blue (Gibco), the total number of cells and the proportion of Trypan blue-positive cells were determined using a Malassez cell.

### Immunofluorescence

N/TERT-1 or primary human KC were seeded on coverslips (12 mm in diameter, 1.5 mm thick; Knittel glass) placed in 24-well plates (2 × 10^4^ cells/well) and 24 h later were treated or not with IMD-006. After the indicated times, cells are washed twice in PBS, then fixed in 3.7% paraformaldehyde (PFA, Electron Microscopy Sciences) for 15 min before neutralizing free aldehydes with NH_4_Cl (Sigma Aldrich) at 50 mM for 2 min. Cells were permeabilized with 0.2% Triton (Sigma Aldrich) then saturated in PBS with 10% FCS, 2% BSA and 0.2% triton for 1 h. Then, the primary antibodies directed against Ki67 (Abcam, reference ab16667 diluted to 1/250) or involucrin (Ab Biotech, reference R0294A diluted to 1/1000) in PBS with 2% BSA and 0.2% Triton were deposited on each slide and left for 2 h. After washing, secondary anti-rabbit (for Ki67, ThermoFisher, reference A-11008, diluted at 1/500) or anti-mouse (for involucrin, ThermoFisher, reference A-11001, diluted at 1/1000) antibodies were applied for 1 h before counterstaining with DAPI (Sigma Aldrich) at 1 μM for 15 min. Coverslips were then mounted on observation slides and stored at −20 °C until observation. Images were acquired with a Nikon 90i optical microscope (for Ki67) or a Leica TCS SP8 confocal microscope (for involucrin), then processed with ImageJ software. The percentage of Ki67 positive nuclei were counted manually and the integrated density of involucrin staining per cell was measured using ImageJ software, on at least 5 images in each independent experiment.

### KC morphology analysis

KC were seeded in 12-well plates (5 × 10^4^ cells/well) and, 24 h later, were treated or not with IMD-006 for 72 h. Then, cells were harvested, washed and their size (Forward Scatter, FSC) and granularity (Side Scatter, SSC) were assessed using an LSR-Fortessa cytometer (BD Biosciences). Data was treated and formatted with FlowJo software.

### IMD-006-NIR uptake assay

KC were seeded in 12-well plates (5 × 10^4^ cells/well) and, 24 h later, were treated or not with the indicated concentrations of IMD-006-NIR for 1 h. Then, cells were harvested, washed and fluorescence was analyzed using an LSR-Fortessa cytometer (BD Biosciences). Data was treated and formatted with FlowJo software.

### Mitochondrial and lysosomal stainings and analyses

N/TERT-1 KC were seeded in 8-well µ-slides (2 × 10^4^ cells/well, Ibidi) for confocal microscopy or in 12-well plates (5 × 10^4^ cells/well) for cytometry analyses, and were treated or not with IMD-006 or IMD-006-NIR. After treatment for the indicated times, cells were washed and stained with MitoTracker Green (100 nM, Invitrogen), MitoSOX™ Red (1 µM, Invitrogen), LysoTracker Red DND-99 (50 nM, Invitrogen) or LysoSensor Green DND-189 (50 nM, Invitrogen) diluted in KC culture medium for 30 min.

After staining, for confocal microscopy, cells were washed and replaced in fresh preheated medium then observed immediately with a Leica TCS SP8 microscope (for capturing of images) or a Zeiss LSM 980 AiryScan2 (for video microscopy), both equipped with a live-cell imaging system allowing the cells to be maintained at 37°C with 5% CO_2_ throughout the image acquisition process. For each experiment, all images were processed in the same manner with ImageJ software. Co-localisation analyses were performed using RGBprofiler and Jacop plug-ins.

For cytometry, after washing, cells were harvested and resuspended in PBS with 1% BSA and fluorescence was analyzed using an LSR-Fortessa cytometer (BD Biosciences). Data was treated and formatted with FlowJo software.

## Supporting information

S1 Fig Protocol of imiquimod (IMQ) treatments of mice. Psoriasis-like lesions were induced on the back skin of Balb/c 8-week-old female mice by application of 80 mg of IMQ daily for 7 days. In parallel, mice were treated with IMD-006 (50 mg/kg) applied topically to the area each day. In some cases, mice were treated with dermoval cream (300 mg/kg) in place of IMD-006. Healthy control mice received a xanthan-based hydrogel in place of IMQ. Daily, the clinical score of animals was assessed based on erythema, thickness and scaling of skin on a scale of 0–4 for each. Histological scoring of skin was performed based on hyperkeratosis, acanthosis, parakeratosis, spongiosis and immune cell infiltrate on a scale of 0–4 for each.
(PPTX)

S2 Fig IMD-006 modulates the infiltration of immune cells in IMQ-induced lesions. CD3^+^ T cells, Ly-6C/Ly-6G^+^ polynuclear neutrophils (PNN), and F4/80^+^ macrophages are immunodetected on skin sections of control, imiquimod (IMQ), and IMQ + IMD-006 treated-mice. Representative images are shown, and staining intensity was quantified. Statistical significance of differences was assessed using a one-way ANOVA with Tukey’s post-test for PNN and Kruskall-Willis with Dunn’s post-test s for T cells and macrophages. N = 6 mice per group.
(PPTX)

S1 Video N/TERT-1 KC were seeded in micro-slide chambers for 24 h before being washed and stained with MitoTracker Green (MT, green) and LysoTracker Red DND-99 (LT, magenta). They had been then imaged for 12 h under physiological conditions using a live-cell imaging system. Supplementary video S1 shows control KC (not treated with the IMD-006 dendrimer during the 24 h incubation in micro-slide chambers).
(AVI)

S2 Video N/TERT-1 KC were seeded in micro-slide chambers for 24 h before being washed and stained with MitoTracker Green (MT, green) and LysoTracker Red DND-99 (LT, magenta). They had been then imaged for 12 h under physiological conditions using a live-cell imaging system. Supplementary video S2 shows KC treated with IMD-006 at 20 µM during the 24 h incubation.
(AVI)

S3 Video N/TERT-1 KC were seeded in micro-slide chambers for 24 h before being washed and stained with MitoTracker Green (MT, green) and LysoTracker Red DND-99 (LT, magenta). They had been then imaged for 12 h under physiological conditions using a live-cell imaging system. Supplementary video S3 shows KC not treated with the IMD-006 dendrimer during the 24 h incubation but to which 20 µM of IMD-006 was added to MT and LT pre-stained cells just before beginning acquisition and remained in the well throughout the 12 h of imaging.
(AVI)

S1 File Raw data set Fig 1. Values behind the means and standard deviations used to build the graphs Figs 1C, 1E and 1F.
(XLSX)

S2 File Raw data set Fig 2. Values behind the means and standard deviations used to build the graphs Fig 2B.
(XLSX)

S3 File Raw data set Fig 3. Values behind the means and standard deviations used to build the graphs Fig 3B.
(XLSX)

S4 File Raw data set Fig 4. Values behind the means and standard deviations used to build the graphs Figs 4A, 4B, 4E and 4F.
(XLSX)

S5 File Raw data set Fig 5. Values behind the means and standard deviations used to build the graphs Figs 5A, 5B, 5E and 5F.
(XLSX)

S6 File Raw data set Fig 6. Values behind the means and standard deviations used to build the graphs Fig 6C.
(XLSX)

S7 File Raw data set Fig 7. Values behind the means and standard deviations used to build the graphs Fig 7C.
(XLSX)

S8 File Raw data set Fig 8. Values behind the means and standard deviations used to build the graphs Fig 8B.
(XLSX)

S9 File Raw data set S2 Fig. Values behind the means and standard deviations used to build the graphs S2 Fig.
(XLSX)

## Abbreviations

DC: dendritic cells
IHC: immunohistochemistry
IL: interleukin
IMQ: imiquimod
KC: keratinocytes
RHE: reconstructed human epidermis
ROS: reactive oxygen species
Th: T helper
