# Supplementary material for: A phosphorous-based dendrimer targets mitochondria and normalizes the keratinocyte proliferation/differentiation balance to improve psoriasis
Source: PLoS One. 2026 Mar 31;21(3):e0343926. doi: 10.1371/journal.pone.0343926 (PMC13038018; doi:10.1371/journal.pone.0343926)
Supplement: S1 Fig — Psoriasis-like lesions were induced on the back skin of Balb/c 8-week-old female mice by application of 80 mg of IMQ daily for 7 days. In parallel, mice were treated with IMD-006 (50 mg/kg) applied topically to the area each day. In some cases, mice were treated with dermoval cream (300 mg/kg) in place of IMD-006. Healthy control mice received a xanthan-based hydrogel in place of IMQ. Daily, the clinical score of animals was assessed based on erythema, thickness and scaling of skin on a scale of 0–4 for each. Histological scoring of skin was performed based on hyperkeratosis, acanthosis, parakeratosis, spongiosis and immune cell infiltrate on a scale of 0–4 for each. (PPTX) [file pone.0343926.s001.pptx]

## Slide 1
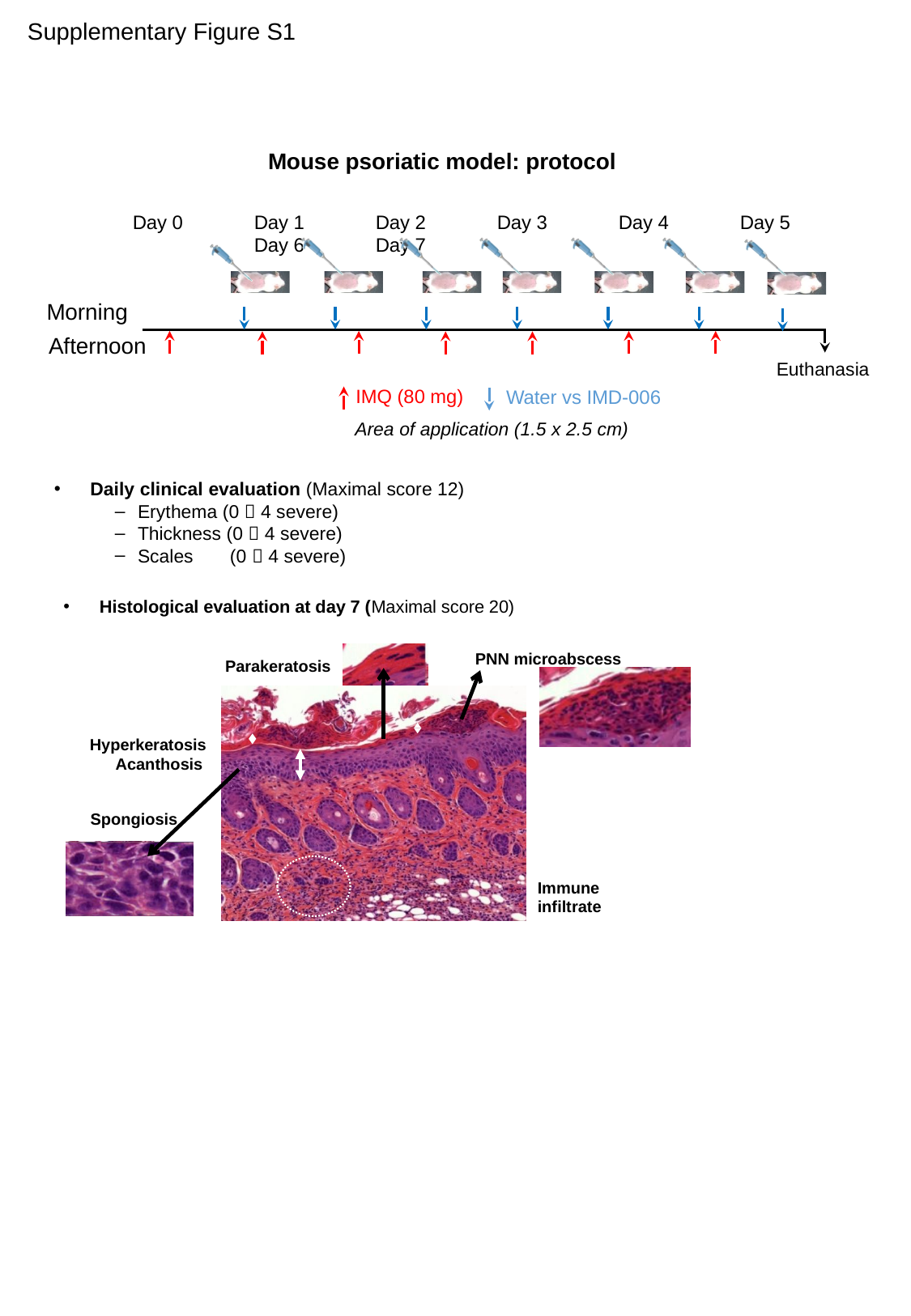

Supplementary Figure S1
Mouse psoriatic model: protocol
Day 0	Day 1	Day 2	Day 3	Day 4	Day 5	Day 6	Day 7
Morning
Afternoon
Euthanasia
IMQ (80 mg)
Water vs IMD-006
Area of application (1.5 x 2.5 cm)
Daily clinical evaluation (Maximal score 12)
Erythema (0  4 severe)
Thickness (0  4 severe)
Scales (0  4 severe)
Histological evaluation at day 7 (Maximal score 20)
PNN microabscess
Parakeratosis
Hyperkeratosis
Acanthosis
Spongiosis
Immune infiltrate
