# Supplementary figures and images for: A phosphorous-based dendrimer targets mitochondria and normalizes the keratinocyte proliferation/differentiation balance to improve psoriasis
Source: PLoS One. 2026 Mar 31;21(3):e0343926. doi: 10.1371/journal.pone.0343926 (PMC13038018; doi:10.1371/journal.pone.0343926)

## Slide 1
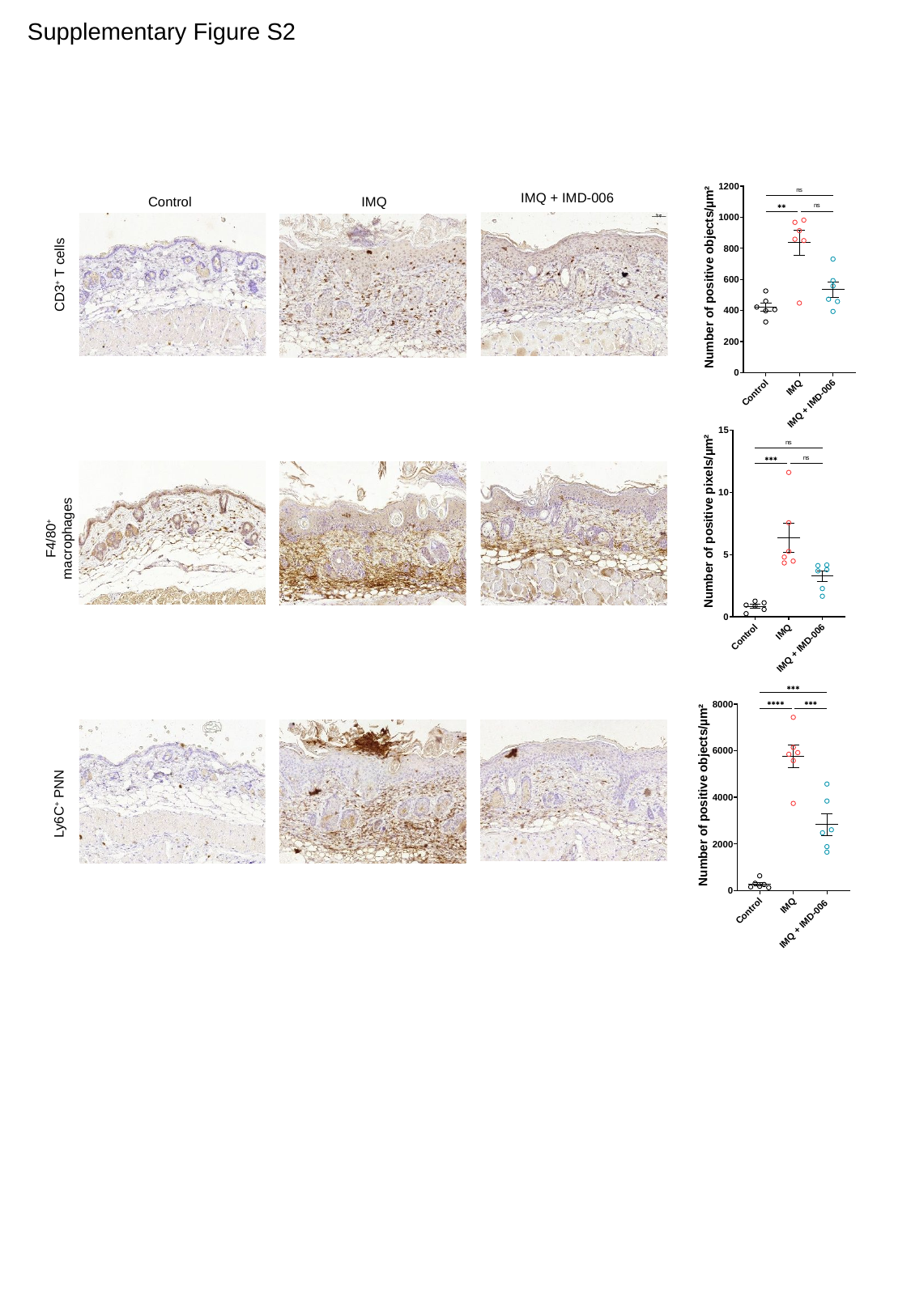

Supplementary Figure S2
IMQ + IMD-006
Control
IMQ
CD3+ T cells
F4/80+ macrophages
Ly6C+ PNN

Supplement: S2 Fig — CD3+ T cells, Ly-6C/Ly-6G+ polynuclear neutrophils (PNN), and F4/80+ macrophages are immunodetected on skin sections of control, imiquimod (IMQ), and IMQ + IMD-006 treated-mice. Representative images are shown, and staining intensity was quantified. Statistical significance of differences was assessed using a one-way ANOVA with Tukey’s post-test for PNN and Kruskall-Willis with Dunn’s post-test s for T cells and macrophages. N = 6 mice per group. (PPTX) [file pone.0343926.s002.pptx]
